# Identification of *Echinococcus granulosus* Strains in Isolated Hydatid Cyst Specimens from Animals by PCR-RFLP Method in West Azerbaijan – Iran

**Published:** 2013

**Authors:** Haleh HANIFIAN, Kambiz DIBA, Khosrow HAZRATI TAPPEH, Habib MOHAMMADZADEH, Rahim MAHMOUDLOU

**Affiliations:** 1 Dept. of Medical Parasitology and Mycology, Faculty of medicine, Urmia University of Medical Sciences, Urmia, Iran; 2 Cellular and Molecular Research center, Urmia University of Medical Seiences, Urmia-Iran; 3 Dept. of surgery, Faculty of medicine, Urmia University of Medical Sciences, Urmia-Iran

**Keywords:** Hydatid cyst, RFLP-PCR, Strain, ITS1

## Abstract

**Background:**

The aim of this study was DNA extraction from protoscolecses of *Echinococcus granulosus* and identification of these strains in West-Azerbaijan Province, north western Iran.

**Methods:**

Thirty one livestock isolates from sheep and cattle were collected from abattoirs of the province. To investigate the genetic variation of the isolates, after DNA extraction by Glass beads-phenol chloroform method; PCR-RLFP analysis of rDNA-ITS1 was performed using three different restriction enzymes of *Taq 1, Rsa 1* and *Alu 1*.

**Result:**

Amplified PCR products for all isolates were 1000bp band which is expected band in sheep strains (G1-G3 complex). The results of RFLP analysis also were the same for all isolates. PCR-RFLP patterns restriction enzymes were identical as follows, Rsa1 bands under UV showed two bands approximately 655bp and 345bp. *Alu*1 bands were as follows: two approximately 800bp and 200bp and *Taq1* did not cut any region and bands were approximately 1000 bp in all samples.

**Conclusions:**

Based on PCR-RFLP patterns of ITS1 fragment produced with endonucleases enzyme digestion in animal isolates, it can be concluded that a single strain of *E. granulosus* (sheep strain or G1-G3 complex) is dominant genotype in this province.

## Introduction

Cystic echinococcosis (CE) is an ancient zoonotic disease caused by the metacestode of the dog tapeworm *Echinococcus granulosus*. The disease is of worldwide importance and it is widespread in eastern regions (1).

In Iran, this disease has brought about many problems in animal husbandry too. Dog, jackal and fox are the definitive host of the parasite and the cyst stage develop in herbivores animals specially sheep, cattle, goat and camel. Lorestan Province of Iran, with its special ecological conditions and on the basis of the previous studies, has a high incidence rate of hydatid cyst in livestock (2).

*Echinococcus granulosus* comprises a number of genetic variants. To date, the former species *E*. granulosus comprises eight genotypes (G1–G3, G6–G9 and G10) and two species, *Echinococcus* equines (G4) and *Echinococcus* ortleppi (G5). This categorization follows closely the pattern of strain variation based on biological characteristics (3).

In previous studies in Iran, sheep (G1-G3) and camel (G6) strains have been identified. These studies have been accomplished in the central and western areas (4–6).

Until now, several researches have been done using molecular methods for identifying the strain varieties of this parasite in Iran but a few number of the samples decreased reliability of the results. Since different strains of *Echinococcus* show different biologic and pathogenic behavior, identification of these strains and their incidence can help recognition of the biologic cycle of the parasite (2).

The aim of this study was DNA extraction from protoscolecses of *Echinococcus granulosus* and identification of these strains in West-Azerbaijan Province, north western Iran.

## Material and Methods

West Azerbaijan Province is located in the North West of Iran, bordering Turkey, Iraq and Nakhchivan, and the provinces of East Azerbaijan, Zanjan and Kurdistan. The province of West Azerbaijan covers an area of 39,487 km^2^, or 43,660 km^2^ including Lake Urmia. In 2006 the province had a population of 3,015,361 (7).

This province with mountainous temperate climate, suitable pastures for traditional animal husbandry and the existence of the migrating tribes, have provided suitable conditions for parasite transfer. The molecular epidemiology of *E. granulosus* strains in West Azerbaijan in domestic herbivores in this study can clarify the circumstances of distribution and variety of strains which culminate in better preventive measures.

### Parasite specimens

During the period of study (June 2010 - September 2010), the parasitic cysts of *E. granulosus* were collected from slaughterhouses of different regions of West Azerbaijan (Urmia, Miandoab, Khoy, Makou, Boukan). Forty three isolates were collected. 16 samples were from cattle and 27 samples were from sheep. Only 31 samples were fertile. Cyst of *E. granulosus* was identified on the basis of presence of protoscoleces.

Protoscoleces were isolated from the fertile cysts. In brief, hydatid fluid was withdrawn from the cyst, cyst wall was excised and protoscoleces were collected by sterilized needle and syringe. The isolated protoscoleces were washed three times by phosphate buffer saline (PBS) (pH 7.2) and preserved in 70% alcohol keeping at 4 ^°^C.

### DNA extraction

Protoscoleces sediments were selected to DNA extraction stage. Equal volumes of packed protoscoleces (about 30-50 µl) were washed twice with sterile distilled water to remove ethanol. Then 300µl lysis buffer (NaCl 0.1M, EDTA 0.01M, Tris- HCl 0.1M, SDS 1%) added to the sediment of each tube. The subsequent DNA extraction process performed in two steps:

-Step one, which was used for disruption of cells and DNA release:

About 300 µl of 0.5 mm diameter glass beads was added to each tube

-Step two:

Thirty (30)µg of proteinase K (Fermentase, Lithuania) was added to each tube containing samples plus 300µl lysis buffer and incubated at 56 °C for one hour. Then, 300µl phenol chloroform was added and centrifuged at 5000 rpm for 5 min. After removing the supernatant to a new tube, chloroform was added prior to shaking and spanning in 5000 rpm for 5 min. Subsequently equal volume of iso-Propanol (Merck, Germany) and 0.1 volume sodium acetate (Merck, Germany) (3M, pH = 5.2) were added to the supernatant, and kept at -20 °C for 1 hour. Next, it was spun 15 min in 14000 rpm and the sediment was rinsed by 300µl 70% ethanol. After spinning 5 min in 5000 rpm and removing ethanol, pellet was dissolved in 50µl deionized water, and stored at -20 °C.

### PCR process

The PCR was performed for amplification of the ITS1 from all DNA samples. The forward (BD1: 5′ GTC GTA ACA AGG TTT CCG TA 3′) and reverse (4S: 5′ ΄- TCT AGA TGC GTT CGA (A/T) GTC GAT G 3′) primers were used. PCR reaction carried out in 25µl, with 0.5µl DNA extraction product, 0.5µl of each primer (forward and reverse), and 12µl *Taq* Master Mix, 10µl DDW and 1.5µl MgCl_2_. The temperature profile was: One cycle of 95°C for 5 min (primary denaturation), followed by 30 cycles of 94 °C for 30 s (denaturation), 55 °C for 45s (annealing), and 72 °C for 45 s (extension), and a final extension 72 °C for 5 min. The extracted DNAs and PCR products of each method were loaded on 1.5% TBE (Tris 0.09M-Borate 0.09M-EDTA 0.02M) agarose gel (Cinnagen, Iran). The gels stained in 1.5µg/ml ethidium bromide in 1lit DDW for 25 minutes. Electrophoresis carried out 1 hour at 80 V. The bands visualized in UV transilluminator and digitally photographed.

### RFLP process

The purified PCR products were digested by three restriction endonucleases *Rsa 1, Alu 1* and *Taq 1*, which were effective on different regions of ITS1; in defined heat and time, as below:

*Alu 1* = (5’ AG/CT 3’), 37^°^, 1h

*Rsa 1* = (5’ GT/AC 3’), 65^°^, 1h

*Taq 1* = (5’ T/CGA 3’), 37^°^, 1h

The sizes of the restricted products were assessed by electrophoresis in 2% (w/v) TBE agarose gel, stained with 0.5µg/ml ethidium bromide.

## Results

**Fig. 1:**
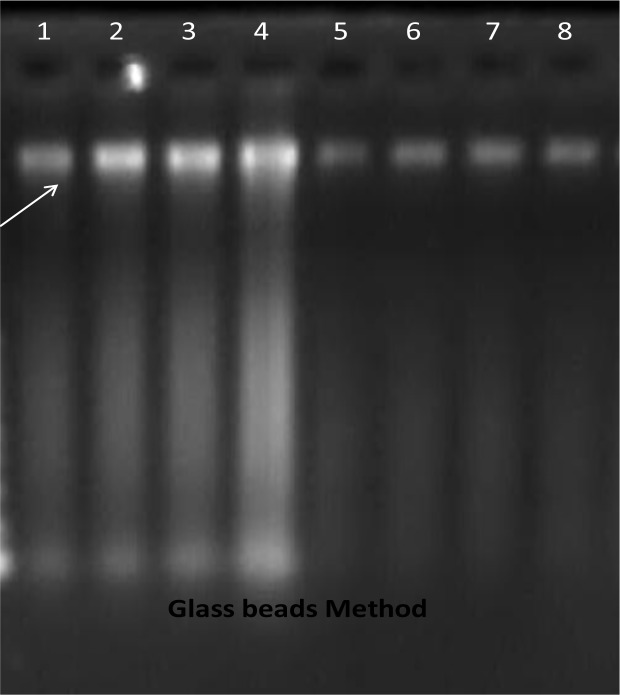
extraction products using Glass beads method (1-4 sheep / 5-8 cattel sampels)

In the present study 43 cyst samples were isolated from domestic livestock slaughtered in different cities of West Azerbaijan province (Urmia, Khoy, Miandoab, Boukan). Ten of them were negative in presence of protoscolecs and 2 of them were infected by Bacteria. The sizes of the bands were the same in all sheep and cattle isolated and were about 1000bp which is similar to the sheep strain (Fig. 1).

RFLP was done on PCR amplified ITS1of rDNA isolated from sheep, cattle using *Taql, Alul* and *Rsal* restriction enzymes. The results of RFLP showed that all of the samples were the same and similar to the sheep strain pattern (*E. granulosus* sensu stricto (G1-G3).

**Fig. 2:**
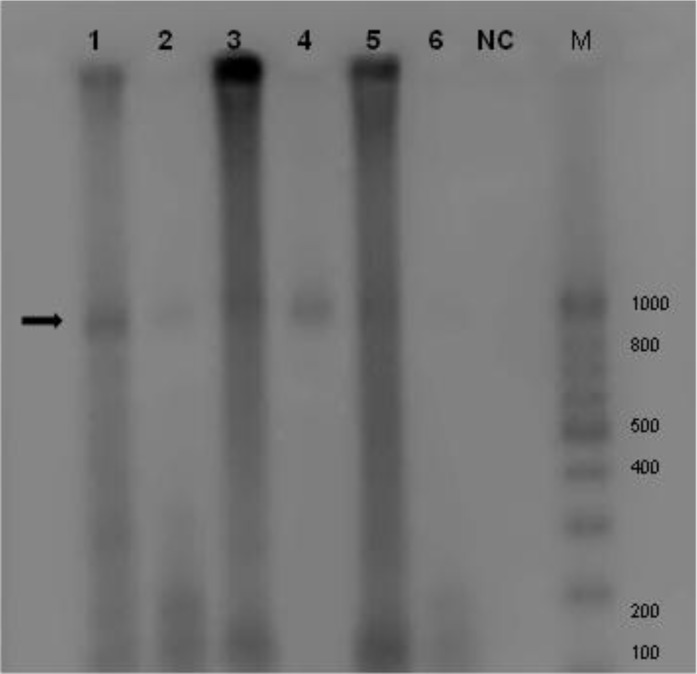
The attained pattern from restricting enzymes on rDNA-ITS1 upon agarose gel; *Taq*1NC: negative control, M: ladder (1-4 sheep/ 5 and 6 cattle)

**Fig. 3:**
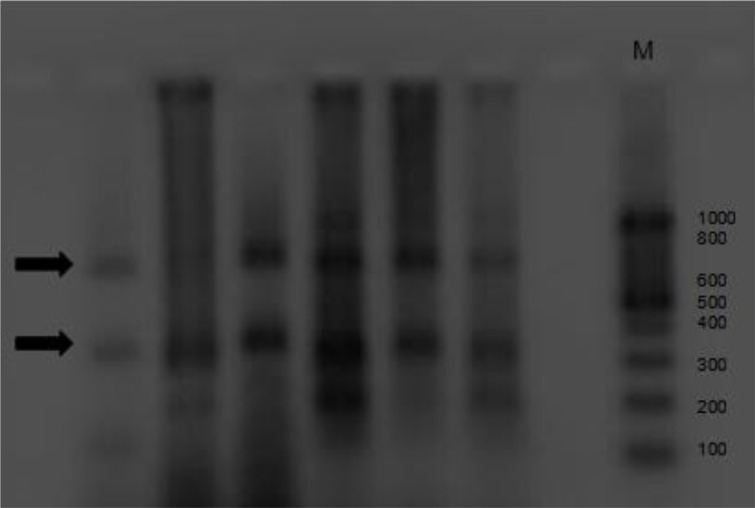
The attained pattern from restricting enzymes on rDNA-ITS1 upon agarose gel; Rsa1

**Fig. 4:**
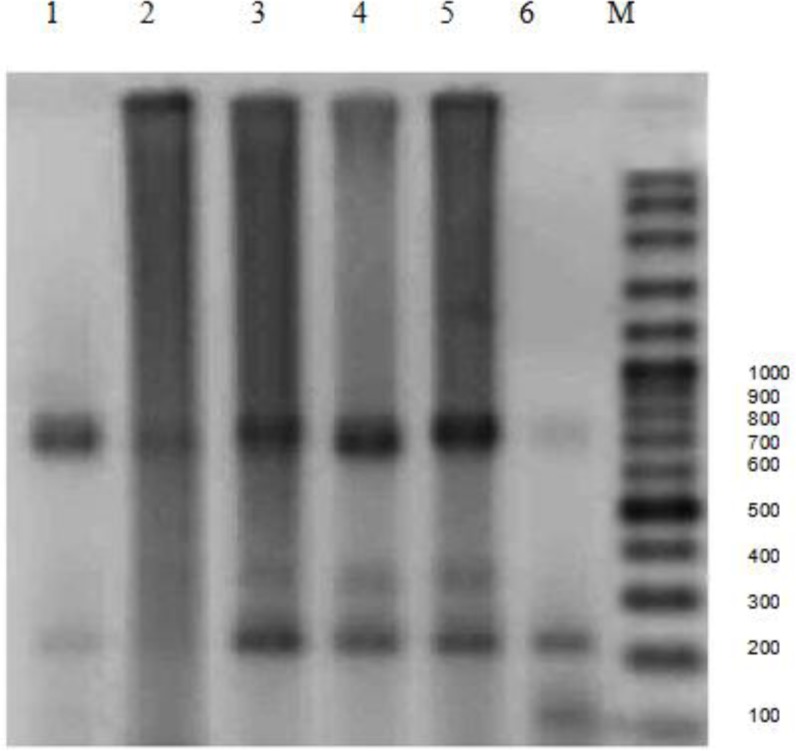
The attained pattern from restricting enzymes on rDNA-ITS1 upon agarose gel; *Alu1*

## Discussion

The taxonomy of *Echinococcus* has not been clear enough due to the taxonomic status of described species and subspecies (12–14). The use of morphology, solely, is not efficient for determination of genetic diversity. Recently, DNA characterization procedures have been used for study of *Echinococcus* variations, as with a few studies in Iran. First of them, was a preliminary study, based on mitochondrial DNA markers, on isolates of human and domestic animals from different areas of the country, indicating the presence of two distinct sheep strain and camel strain in Iran (6). In another study, using PCR-RFLP method on ITS1 and also morphological factors, the sheep strain was the most common genotype in Iran.

Despite geographically, Iran is localized in a high endemicity region, data about the prevalence of CE are still limited. Contamination rate of hydatid cyst in Ardebil province (North West of Iran) as follows: in cattle 16.3%, buffalo 1.6%, sheep 33.8% and goat 5.8%. In other study done in Lorestan, Ilam, Kermanshah and Azerbaijan provinces, the contamination rate was reported in sheep 11.1%, cattle 16.4%, buffalo 12.4% and goat 6.3% (4, 8–11).

This issue in endemic regions such as Iran, that there is more than one species of intermediate host and also there is the possibility of interaction between cycles of transmission. The design and performing of control program should be supported by such information and, in particular, which cycles of transmission are risks to human health (12–14).

The RLFP pattern resulted from the effect of the restriction enzymes indicated the presence of G1 (G1-G3 complex) in West Azerbaijan province that is similar to the results of Bowels and McManus (15) Bhattacharya et al. (16), and Villalobos et al. (17). In different studies done in various provinces in Iran, using similar method to this study, G1 genotype introduced as dominant variant of *Echinococcus granulosus* (2, 4, 18).

Amplified PCR products for all isolates were 1000bp band which is expected band in G1. The results of RFLP analysis also were the same for all isolates. PCR-RFLP patterns restriction enzymes were identical as follows, *Rsa1* bands under UV showed two bands approximately 655bp and 345bp. *Alu1* bands were as follows: two approximately 800bp and 200bp and *Taq1* did not cut any region and bands were approximately 1000bp in all samples. Based on PCR-RFLP patterns of ITS1 fragment produced with endonucleases enzyme digestion in animal isolates, it can be concluded that the important strain of *E. granulosus* is G1 which categorizes in G1-G3 complex, is dominant genotype in this province.

The most prevalent genotype in many countries of Middle East area, including our country has been reported as G1 genotype (10, 19–21) in both of human and animals. Based on the results of this study and other studies reported from our country and Mediterranean area such as Rokni's comprehensive study (22) conducted on 16 isolates of *E. granulosus* from domestic animals including sheep, goats, cattle, and camels, by the method of DNA nucleotide and predicted amino acid sequence variation within regions of the mitochondrial cytochrome oxidase I (COI) and NADH dehydrogenase subunit I (NDI) G6 and G1 genotype reported, it can be suggested that G1 or Sheep genotype is the most important variant of *E. granulosus* and this finding should be taken into account during the planning and implementing *E. granulosus* control programs in our region.

## Conclusion

Existence of *E. granulosus* G1 (sensu strict G1-G3) similar to other provinces in Iran shows that this region is one of the isolated regions in aspect of entering livestock products so health education for tribes and shepherd can prevent the spread of the disease in livestock and its economic loss in native residents.

However, this subject required more detailed comparative studies in endemic regions even in our province again, with different and modified methods in order to more comprehensive results because PCR-RFLP is not capable to differentiate G1-G3 from each other (23) So we recommend examination via gene sequencing using nad1 and cox1 for better identification of strains.
